# Supplementary material for: Pan-proteome regulation of marine Synechococcus nutrient stress plasticity
Source: mBio. 2026 Oct 14;17(11):e02247-26. doi: 10.1128/mbio.02247-26 (PMC13753778; doi:10.1128/mbio.02247-26)
Supplement: Supplementary file 1 [file mbio.02247-26-s0001.pdf]

Supplementary Information Figures:

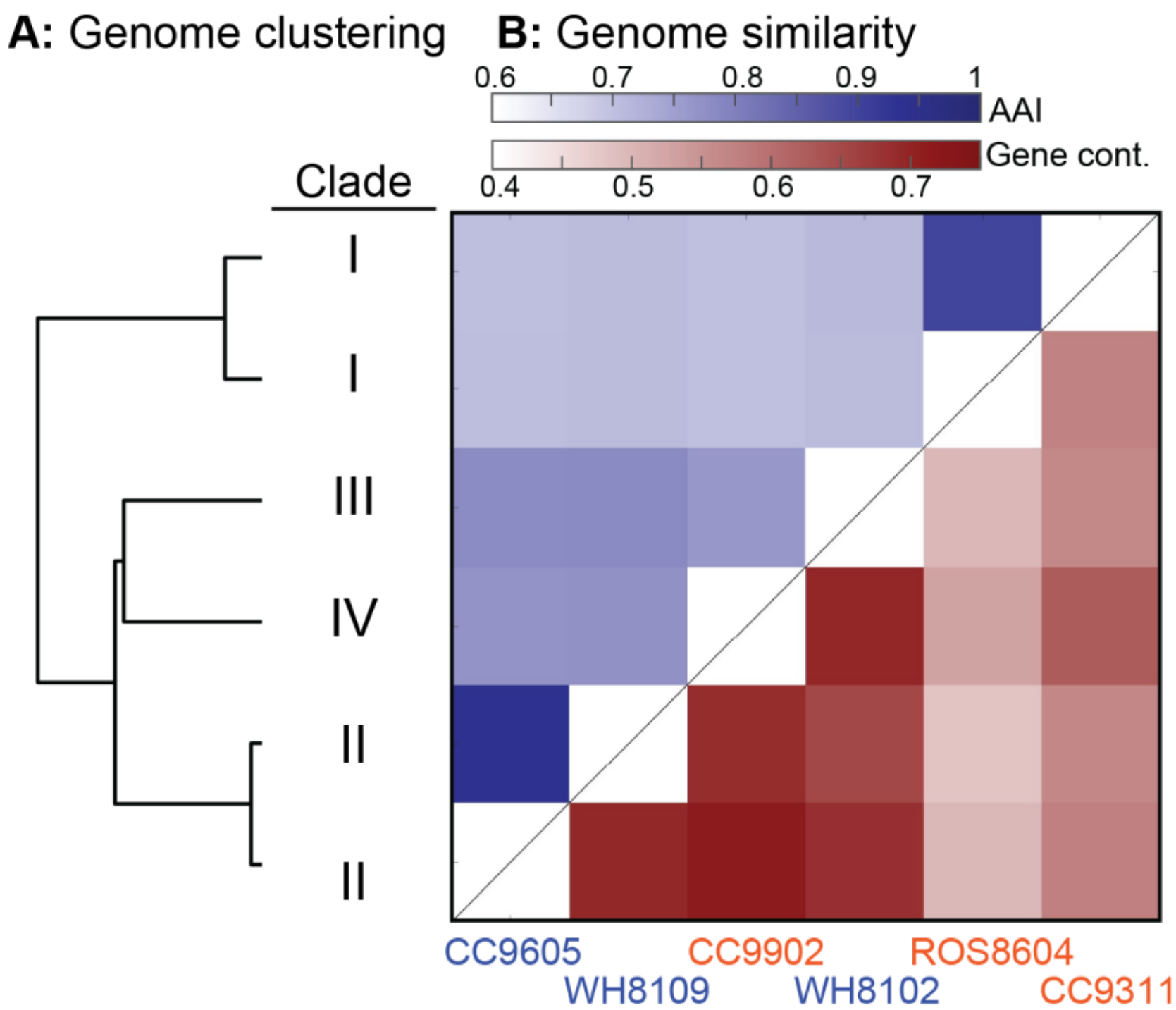

Figure S1: Synechococcus genome similarity. A. Hierarchical clustering of whole genome amino-acid identity (AAI) and associated clade membership. **B.** Pair-wise genome similarity based on AAI (top, white-blue scale) and number of shared genes (bottom white-red scale). The gene content similarity is estimated using the Jaccard index on presence-absence of orthologous genes (using Cyanorak classification). Strain names are color coded according to isolation environment (blue = oligotrophic, orange = mesotrophic).

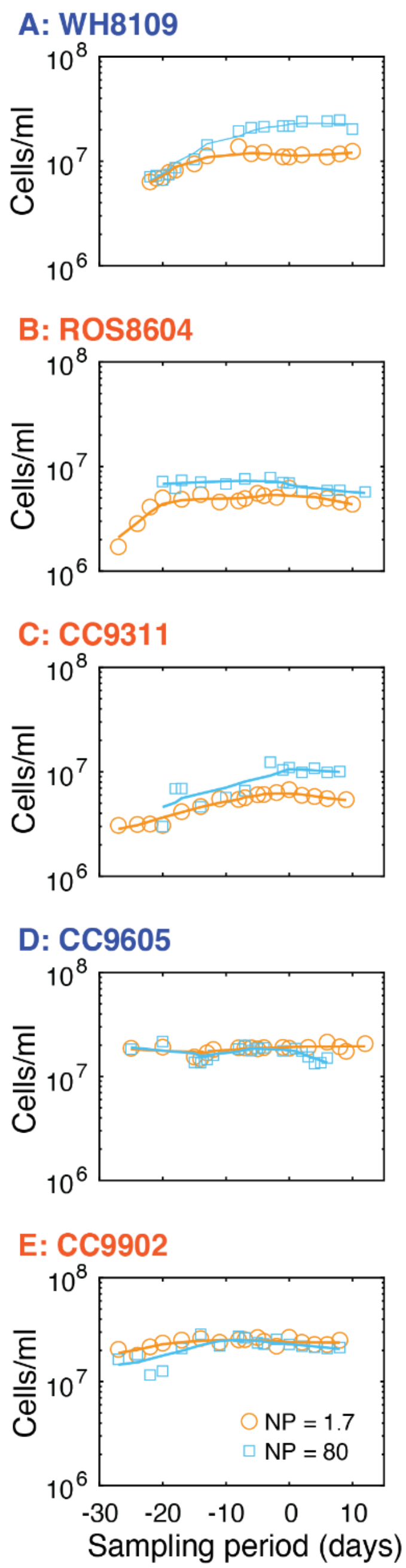

Figure S2: Chemostat cell abundances. The temporal dynamics of *Synechococcus* cells show each chemostat reaching equilibrium after approximately 30 days. Day 0 represents the first time point for cell quotas and proteomic sampling. Each chemostat was sampled 5 to 6 times. Strain names are color-coded according to isolation environment (blue = oligotrophic, orange = mesotrophic).

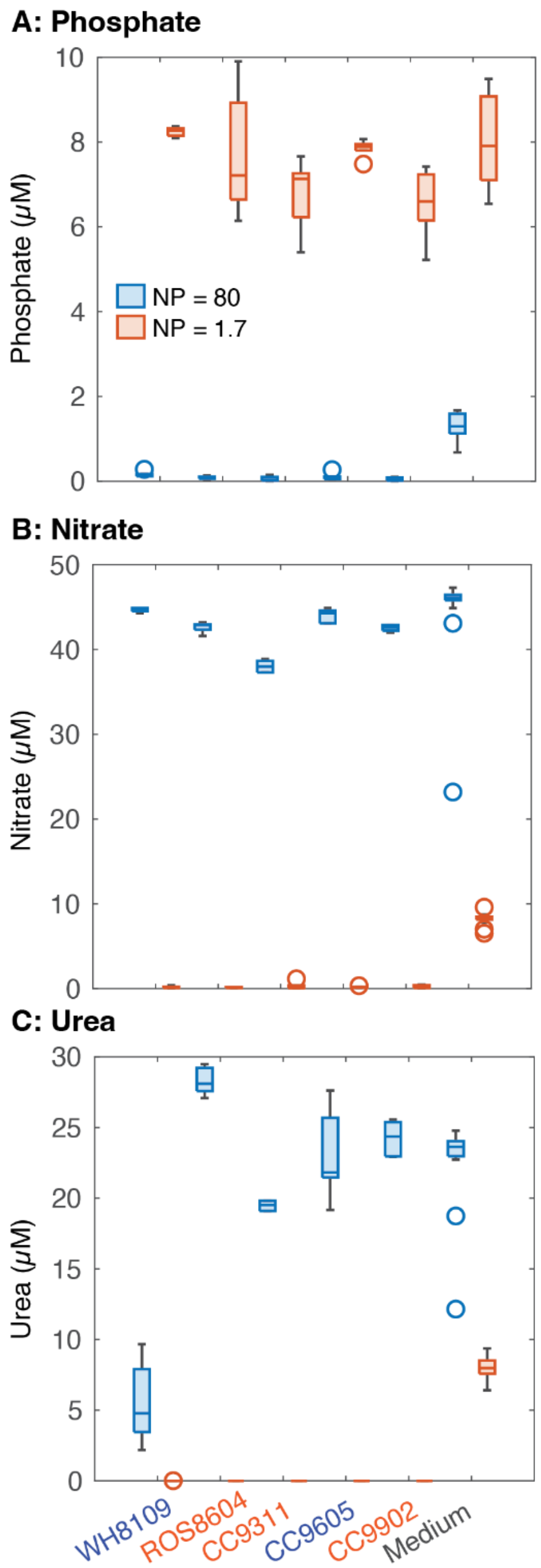

Figure S3. Nutrient concentrations in chemostats and input media. Note that urea contains two nitrogen atoms and thus provided in half the concentration of nitrate. Nutrient concentrations are measured in the input media as well as during each sampling timepoint. The analysis shows that phosphate is completely depleted when the $N{:}P_{input}$ is equal to 80. Nitrate and urea are completely depleted when the $N{:}P_{input}$ is equal to 1.7. Hence, the treatments led to strong single element nutrient limitation. Strain names are color-coded according to isolation environment (blue = oligotrophic, orange = mesotrophic).

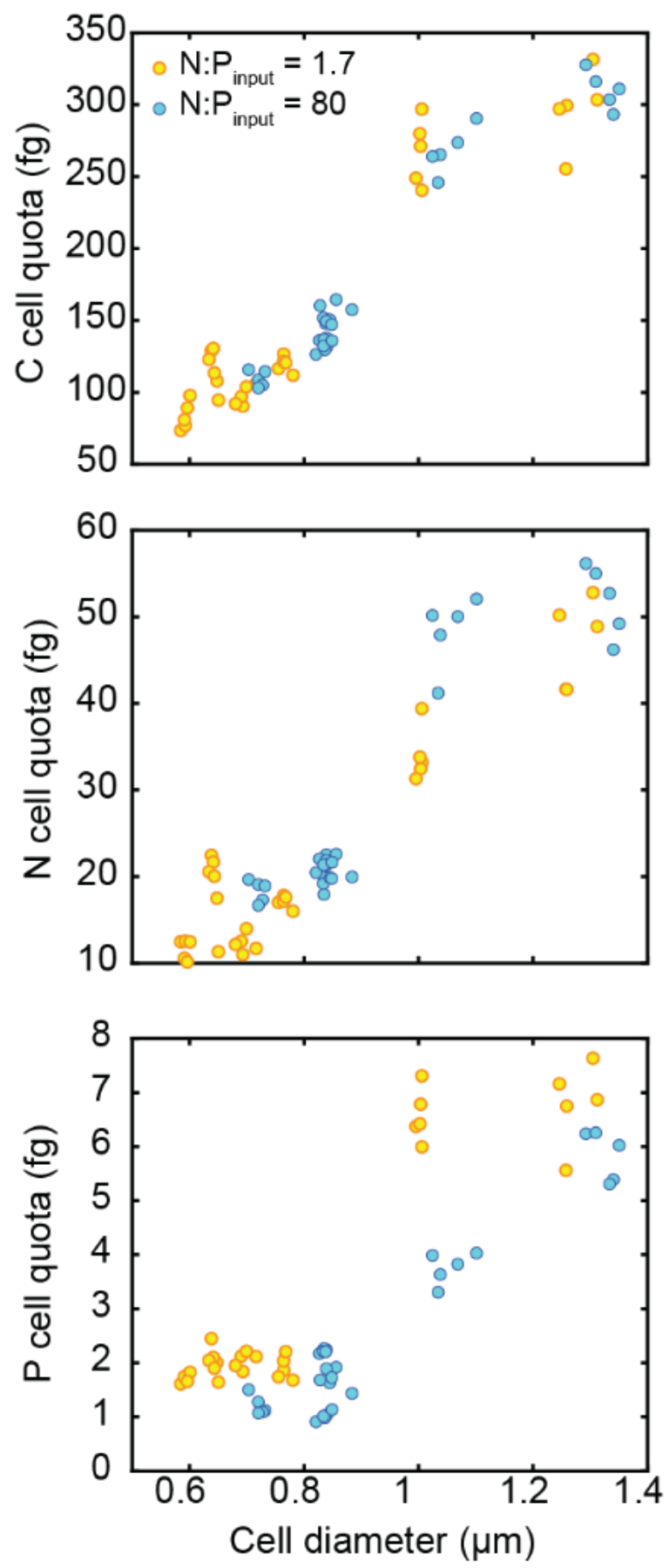

Figure S4. Cell-size dependence of elemental quotas.

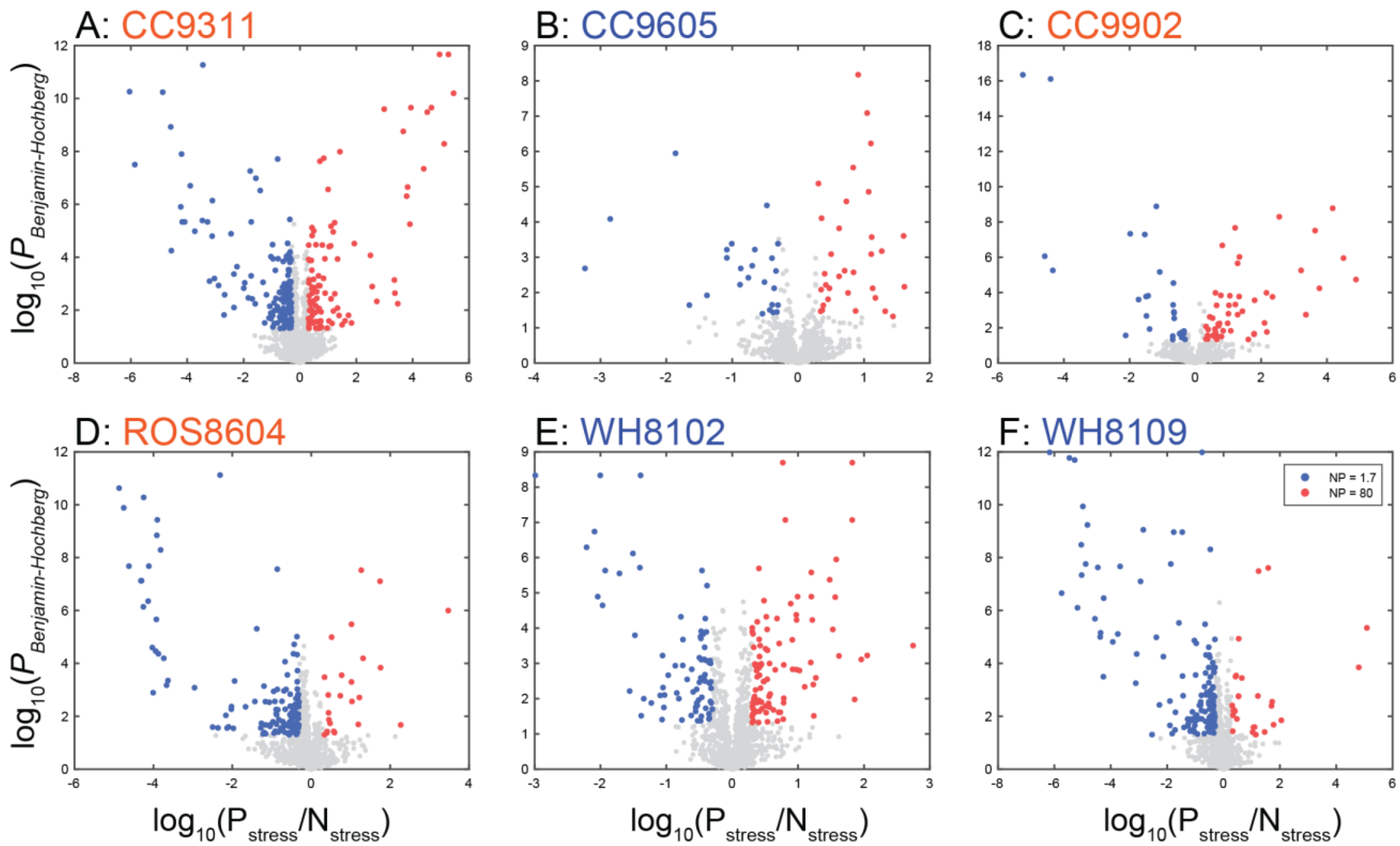

Figure S5. Strain-specific proteome expression responses. The volcano plots show the log-normalized PAI for $N{:}P_{input}$ = 80 divided by $N{:}P_{input}$ = 1.7 on the x-axis for each strain. The y-axis shows the Benjamin-Hochberg false-discovery test for a t-test between each protein expression by treatment. Grey points represent a difference in PAI less than 2X or a $P_{Benjamin\text{-}Hochberg} > 0.05$.. See Table S2 for the full statistics. Strain names are color-coded according to isolation environment (blue = oligotrophic, orange = mesotrophic).

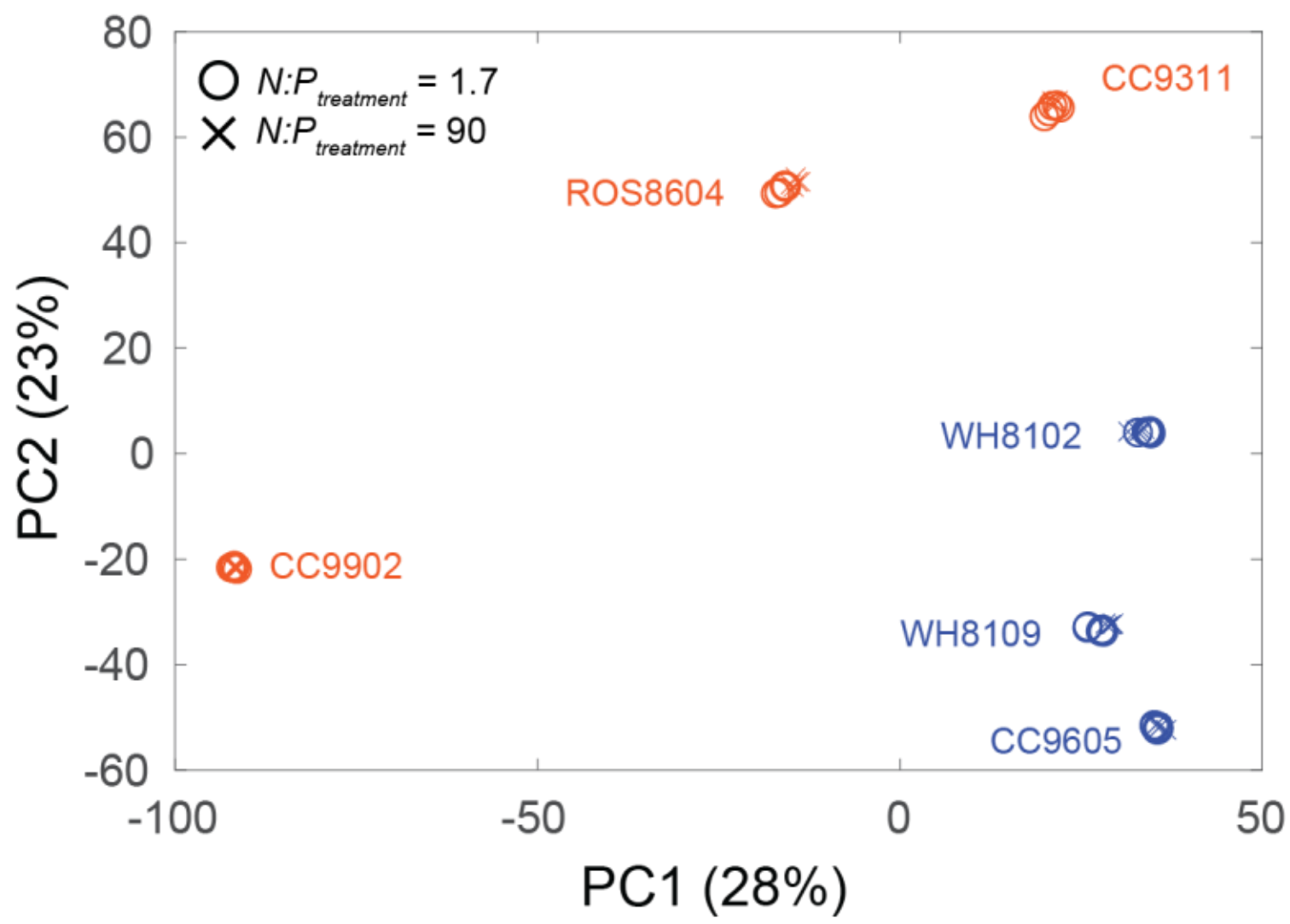

Figure S6. Ordination of core protein expression profiles. Principal components analysis was done on log10 transformed expression values of core proteins (i.e., shared among all strains). Strain names are color-coded according to isolation environment (blue = oligotrophic, orange = mesotrophic).

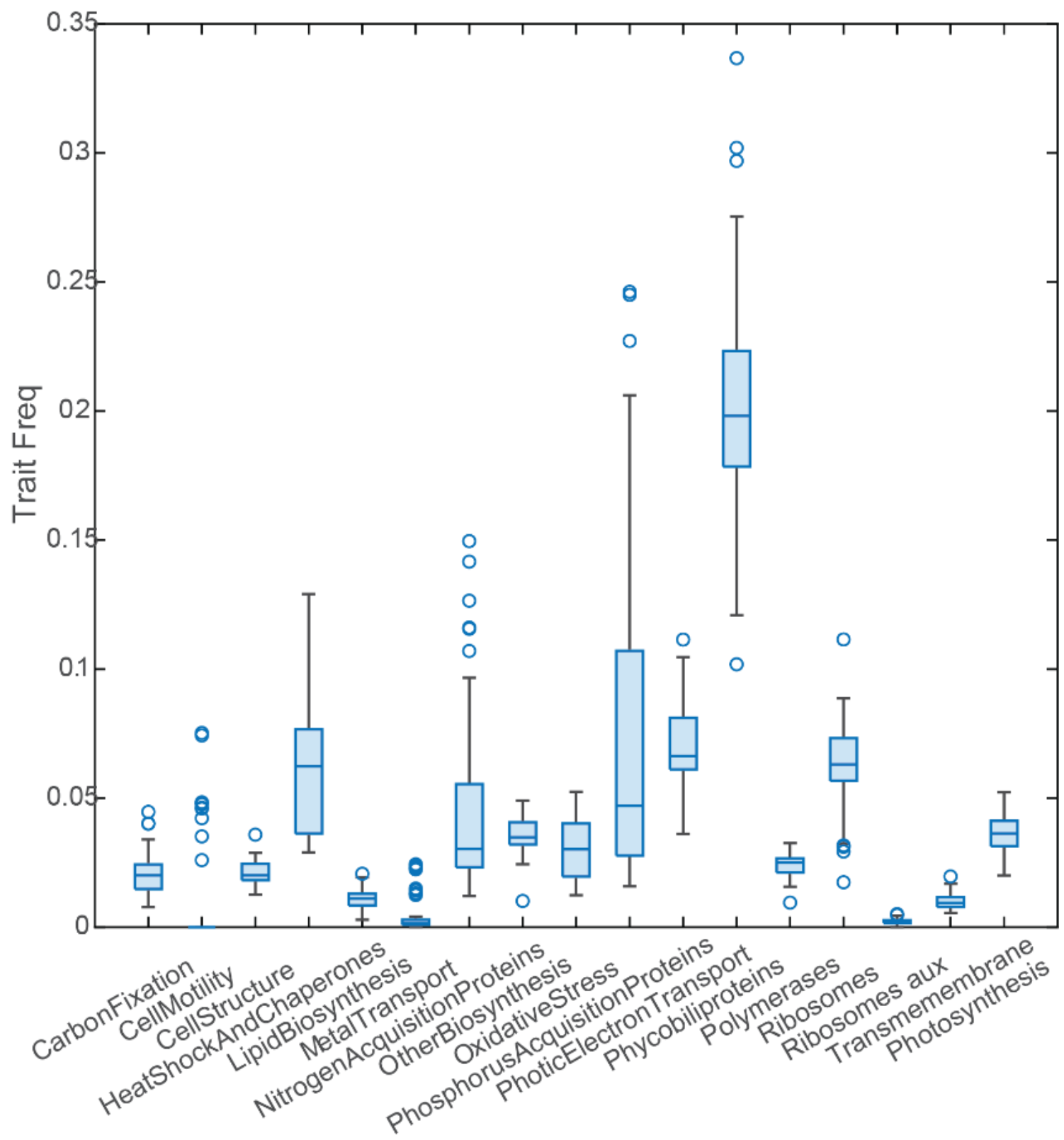

Figure S7. Trait expression across strains. Trait expression across 19 traits. The expression of each trait was done by binning PAI for all genes associated with each trait (see Table S1 for list of genes).
