# Supplementary material for: Pan-proteome regulation of marine Synechococcus nutrient stress plasticity
Source: mBio. 2026 Oct 14;17(11):e02247-26. doi: 10.1128/mbio.02247-26 (PMC13753778; doi:10.1128/mbio.02247-26)
Supplement: Supplementary file 2 [file mbio.02247-26-s0002.docx]

**Table S1: Summary of genomically encoded traits in *Synechococcus***. The table displays the presence of genes associated with various traits in each of the six analyzed *Synechococcus* strains. The table is organized by CyanoRak ortholog IDs.

**Table S2: Log10 normalized protein expression levels in each strain and treatment.** The table displays the log10 normalized expression level for each protein in each strain and replicate within treatment. Genbank, locus tag, and CyanoRak IDs are also listed.

**Table S3: Observed protein expression levels in each strain and treatment.** The table displays the observed expression level for each protein in each strain and replicate within treatment. Genbank, locus tag, and CyanoRak IDs are also listed.

**Table S4: Statistical analyses of difference in protein expression by treatment and strain.** The table summarizes the t-test and post hoc analysis of protein expression by strain. ‘lfc’ **represents log fold change, ‘P_ttest’ is the uncorrected significance, ‘BH’ is the Benjamini–Hochberg post hoc correction** for multiple testing, and ‘BHsig’, ‘BHup’, ‘BHdn’ represents whether the protein is significantly regulated including ‘up’ or ‘down’ after post hoc testing.

**Table S5: Summary of ANOVA test for difference in cell quotas and ratios by strain and treatment**. The first column list coefficients (‘coeff’) in the ANOVA model including the partial effect of strain (n=6) and N:P_input_ ratio (n=2) as well as the residual (‘Error’) and total. Each additional column lists the sum-of-squares (XX_SSQ) and probability (XX_prob) of coefficients being different from zero. The analysis covers cell size, C:N, C:P, N:P, carbon (Qc), nitrogen (Qn), and phosphorus (Qp) quotas.

**Table S6: Summary of the multivariate PERMANOVA analysis.** This analysis is done using Adonis in the R package ‘vegan’. The first column lists the independent variables for strain and N:P_input_ together with the residual. Each additional column represents the input data including core gene expression log10 normalized, core gene expression without normalization, all gene expression log10 normalized and all gene expression without normalization. There are no interactive effects in the model.

**Table S7: Biogeochemical observations from each chemostat organized by strain and treatment.**

**Table S8: List of protein expression files and their associated strain, treatment, and sample day.**
